# Cellular Sources and Regional Variations in the Expression of the Neuroinflammatory Marker Translocator Protein (TSPO) in the Normal Brain

**DOI:** 10.3390/ijms19092707

**Published:** 2018-09-11

**Authors:** Calina Betlazar, Meredith Harrison-Brown, Ryan J. Middleton, Richard Banati, Guo-Jun Liu

**Affiliations:** 1 Australian Nuclear Science and Technology Organisation, New Illawarra Road, Lucas Heights, NSW 2234, Australia; meredith.harrisonbrown@sydney.edu.au (M.H.-B.); rym@ansto.gov.au (R.J.M.); 2 Discipline of Medical Imaging & Radiation Sciences, Faculty of Medicine and Health, Brain and Mind Centre, University of Sydney, 94 Mallett Street, Camperdown, NSW 2050, Australia

**Keywords:** translocator protein, positron emission tomography, neuroinflammation, mitochondria, immunohistochemistry

## Abstract

The inducible expression of the mitochondrial translocator protein 18 kDa (TSPO) by activated microglia is a prominent, regular feature of acute and chronic-progressive brain pathology. This expression is also the rationale for the continual development of new TSPO binding molecules for the diagnosis of “neuroinflammation” by molecular imaging. However, there is in the normal brain an ill-defined, low-level constitutive expression of TSPO. Taking advantage of healthy TSPO knockout mouse brain tissue to validate TSPO antibody specificity, this study uses immunohistochemistry to determine the regional distribution and cellular sources of TSPO in the normal mouse brain. Fluorescence microscopy revealed punctate TSPO immunostaining in vascular endothelial cells throughout the brain. In the olfactory nerve layers and glomeruli of the olfactory bulb, choroid plexus and ependymal layers, we confirm constitutive TSPO expression levels similar to peripheral organs, while some low TSPO expression is present in regions of known neurogenesis, as well as cerebellar Purkinje cells. The distributed-sparse expression of TSPO in endothelial mitochondria throughout the normal brain can be expected to give rise to a low baseline signal in TSPO molecular imaging studies. Finally, our study emphasises the need for valid and methodologically robust verification of the selectivity of TSPO ligands through the use of TSPO knockout tissues.

## 1. Introduction

The translocator protein 18 kDa (TSPO), previously named peripheral benzodiazepine binding site/receptor, is an outer mitochondrial membrane protein and long posited obligatory “translocator” of cholesterol on which steroidogenesis, and thus life, critically depends [1,2]. The creation and long-term observation of viable TSPO knockout mouse models, however, has helped to modify this earlier conception [3]. TSPO is now emerging as a component of mitochondrial bioenergetics that regulates a broader cellular stress response and is likely to include, among others, the energy-dependent process of steroidogenesis [4,5]. Much of the interest in TSPO in the central nervous system has been based on the observation that injury or progressive disease induces a marked upregulation of TSPO in mitochondria of microglia upon their transition from a resting to an activated state [2,6]. The subsequent development of radioligands for TSPO has given rise to a field of molecular imaging aimed at detecting activated microglia, often generically referred to as “neuroinflammation” in neurological disorders [7,8,9,10,11,12,13,14] and also psychiatric illnesses [15,16,17,18,19,20]. The term “neuroinflammation” has become a generic term that does not distinguish between a local immune response and microglial activation in the wake of neural or other injury [21].

However, it has long been known that a low-level, constitutive TSPO expression also occurs in the normal healthy brain. Earlier studies of TSPO expression in the rodent brain using autoradiography with prototypical TSPO ligands, such as [^3^H]PK11195, revealed increased TSPO binding sites in the olfactory bulb, particularly the lateral olfactory tracts [22,23], the ependyma and choroid plexus of the ventricular system [24,25], as well as the cerebellum [23,26]. The distribution of these binding sites has also been demonstrated in human tissue [27]. At the cellular level, TSPO binding sites have also been observed along blood vessels and in perivascular cells [6,28,29]. The presence of TSPO binding sites in the vascular endothelium has led to proposed refinements of the quantification of TSPO positron emission tomography (PET) imaging in order to account for this additional source of signal [30,31,32,33,34,35,36].

The immunohistochemical detection of constitutive and induced TSPO expression in brain tissue or brain cells has so far not been conclusive. TSPO has been located in cell organelles other than mitochondria [37,38,39], variably in some but not all vascular endothelial cells [30,34,40,41,42] and inconsistently, in astrocytes [43,44,45] and neurons [43,46]. One reason for the inconsistencies in the reported tissue distribution of TSPO, a protein found ubiquitously in all tissues, has been the difficulty to unequivocally establish the specificity of immunohistochemical TSPO antibody stains in the absence of a control tissue known to be without any TSPO expression.

The current study establishes the constitutive distribution of TSPO in the normal mouse brain using a TSPO antibody, the specificity of which has been confirmed in a viable TSPO knockout model [3]. The reported regional and cellular distribution data are important for the interpretation of the increasing number of molecular imaging studies using TSPO ligands, for most of which in vivo selectivity remains to be established. Finally, we report not only the presence of TSPO in neurogenic niches but also in a defined subpopulation of end-differentiated cerebellar neurons that should stimulate research into additional functions of TSPO.

## 2. Results

### 2.1. Regional Distribution

**Figure 1:**
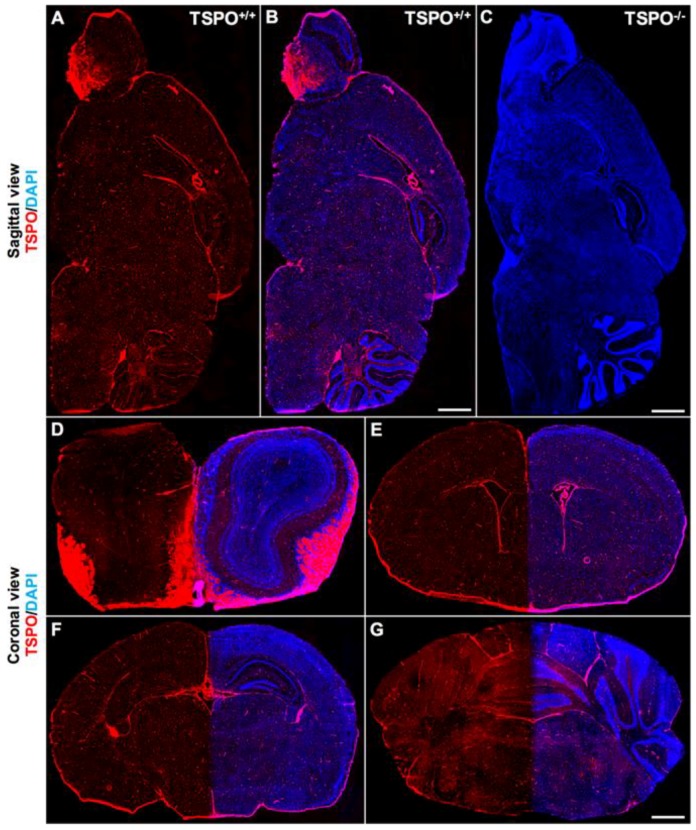
Global immunofluorescence overview of TSPO (translocator protein 18 kDa) expression across major brain regions. (**A**) Sagittal section from a wildtype mouse (TSPO^+/+^) demonstrates widespread TSPO expression (red) across all brain regions using a specific antibody against TSPO (anti-PBR, ab109497). (**B**) Sagittal section from a wildtype mouse with TSPO expression in red and 4’,6-diamidino-2-phenylindole (DAPI) in blue highlighting structural features. Strong TSPO immunoreactivity is present in the olfactory bulb, the choroid plexus and the ependyma of the ventricular system, and cerebellum. (**C**) Sagittal section from a TSPO knockout mouse (TSPO^−/−^) confirming the absence of TSPO expression (DAPI in blue). (**D**) Coronal view of the olfactory bulb demonstrates strong TSPO expression in the olfactory nerve layers and glomeruli, and concentrated expression in the subependymal zone. (**E**) TSPO expression is present in the subventricular zone and the ependymal cells of the ventricles and choroid plexus. (**F**) Coronal view of the hippocampal region with discernible TSPO expression observed in the dentate gyrus. (**G**) Cerebellar/brainstem TSPO expression is strongly present in the molecular layer of the cerebellar cortex and fiber tracts of the brainstem. Scale bars = 500 µm.

Immunohistochemistry was used to determine the precise distribution of TSPO expression in the normal mouse brain across all major brain regions and to delineate the cell types where TSPO expression was observed. The specificity of the TSPO antibody used here (#ab109497, Abcam) has been confirmed through the global TSPO knockout mouse model [3], also demonstrated in this study (Figure 1C). To obtain the distribution of TSPO expression across the entire brain, whole tissue scans of sagittal brain sections were acquired. A sparse, distributed, punctate immunohistochemical TSPO stain was observed throughout the brain parenchyma. More abundantly, highly expressed TSPO was observed in the olfactory bulb (particularly the lateral regions), the ependyma of the ventricular system, and the choroid plexus (Figure 1A,B). The cerebellum also had zones of relatively higher TSPO expression than other brain regions (Figure 1A,B). In general, TSPO immunostaining was observed to a greater degree in white matter regions across the brain compared to grey matter (Figure 1A,B). An absence of TSPO immunoreactivity in the TSPO knockout mouse brain is demonstrated in Figure 1C. Coronal brain sections also revealed salient TSPO expression in the subependymal zone of the olfactory bulb (Figure 1D), the subventricular zone (Figure 1E), the dentate gyrus of the hippocampus (Figure 1F), and the molecular layer of the cerebellar cortex (Figure 1G). Similar findings were also demonstrated with DAB (3,3′-diaminobenzidine) immunostaining using the same TSPO antibody (refer to Supplementary Figure S1).

### 2.2. Cellular Distribution

**Figure 2:**
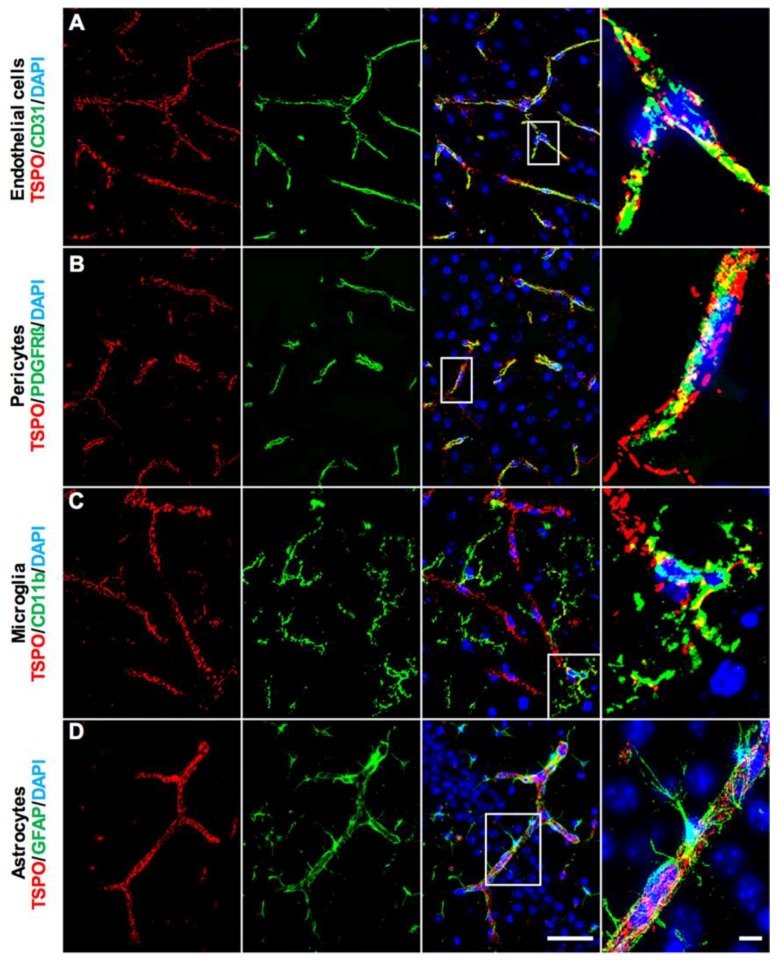
TSPO expression in major cell types of the normal mouse brain. (**A**) Double immunofluorescence staining for TSPO (red) demonstrates punctate staining which colocalizes strongly with CD31+ vascular endothelial cells (green). This is observed in larger blood vessels, smaller arterioles and venules, as well as capillaries. Punctate staining is indicative of mitochondrial localization. (**B**) TSPO expression (red) and PDGFRβ+ pericytes/smooth muscle cells (green) colocalize on larger blood vessel walls. (**C**) No clearly discernible TSPO expression (red) is observed in CD11b+ microglia (green). (**D**) TSPO expression (red) is not observed in GFAP+ astrocytes (green). Perivascular astrocytic endfeet and processes surround CD31+ endothelial cells of blood vessels, where TSPO strictly colocalizes. Scale bars = 20 µm, 10 µm for box insets.

Closer examination of the TSPO immunostaining across brain regions confirmed TSPO staining in the neurovascular niche. More specifically, TSPO expression strongly colocalized with CD31+ vascular endothelial cells across the brain (Figure 2A). The punctate staining pattern of TSPO along blood vessels is consistent with the mitochondrial localization of TSPO within cells. TSPO expression was observed throughout the vasculature, ranging from larger blood vessel walls, to smaller capillaries (refer to Supplementary Figure S2 for more images and DAB immunostaining). Because of the close association between pericytes/smooth muscle cells and blood vessels, we also investigated whether TSPO expression was present in this compartment of the neurovasculature. While some TSPO immunoreactivity colocalized with PDGFRβ+ pericytes, this observation was predominantly restricted to larger blood vessels and TSPO expression mostly follows an endothelial staining pattern beyond colocalization with PDGFRβ (Figure 2B). No clearly discernible TSPO expression was observed in CD11b+ microglial cells across the normal brain (Figure 2C). Perivascular microglia were also immunonegative for TSPO. We also generated high magnification 3D z-stack projections of microglial and TSPO colocalization, which confirmed an endothelial localization of TSPO immunoreactivity, rather than any staining in microglial somata or processes (refer to Supplementary Video S1). High-resolution microscopy of astrocytes and their perivascular endfeet revealed that TSPO is strictly colocalized with the endothelial marker CD31 and is not found in the soma or processes of astrocytes (Figure 2D). Mature neurons were also examined for TSPO expression, though no clearly discernible immunostaining was observed in NeuN+ neurons across the brain (refer to Supplementary Figure S3). These results suggest that the major cell type giving rise to the low level, constitutive TSPO expression in the brain parenchyma are vascular endothelial cells.

**Figure 3:**
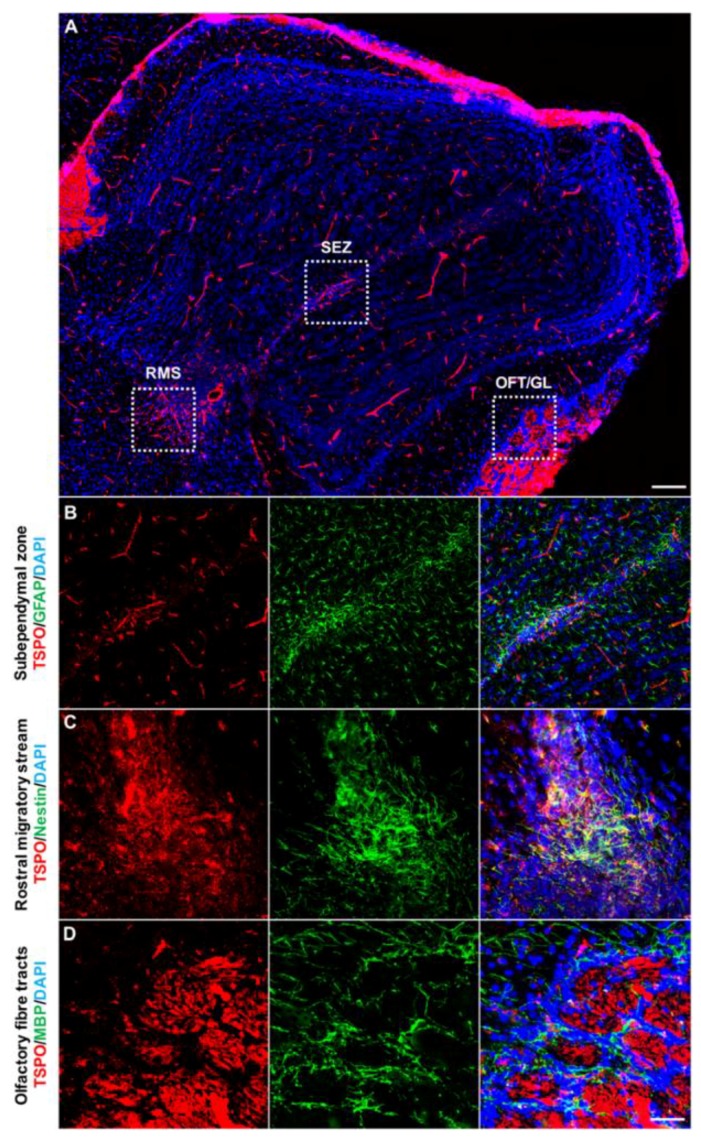
TSPO expression across the olfactory bulb in the normal mouse brain. (**A**) Sagittal view of TSPO expression (red) across the olfactory bulb. Structural features of the region highlighted with DAPI (blue). (**B**) Concentrated TSPO expression (red) is observed in the subependymal zone, which houses a neural stem cell niche derived from the rostral migratory stream (RMS). TSPO colocalizes at low levels with GFAP+ (green) cells. (**C**) TSPO is present at the base of the RMS, entering into the olfactory bulb. Here, TSPO strongly colocalizes with Nestin+ (green) cells. (**D**) TSPO expression (red) is also observed in the olfactory nerve layer and glomerular layer, though does not colocalize with MBP+ oligodendrocytes (green) of the region. Scale bars = 300 µm for (**A**), 20 µm for (**B**–**D**). RMS = rostral migratory stream, SEZ = subependymal zone, OFT = olfactory fibre tracts, GL = glomerular layer.

The olfactory bulb, which houses a subpopulation of neural progenitors and newly formed neurons derived from the rostral migratory stream (RMS), demonstrated significant TSPO immunoreactivity in the subependymal zone, the RMS, the glomerular layer and the olfactory nerve layer (Figure 3A). In the subependymal zone, TSPO colocalized with GFAP positive cells at low levels, likely reflecting TSPO expression in certain subtypes of neural progenitors of this region (Figure 3B). The olfactory region of the RMS, entering into subependymal zone of the olfactory bulb, was also positive for TSPO, colocalizing with Nestin+ neural stem/progenitor cells (Figure 3C). The fiber tracts of the olfactory bulb demonstrated the highest TSPO immunoreactivity comparable to the ependymal layer and choroid plexus in the brain. Here, TSPO was restricted to the axon bundles of the glomerular layer and the olfactory nerve layer, rather than MBP+ oligodendrocytes of the region (Figure 3D).

**Figure 4:**
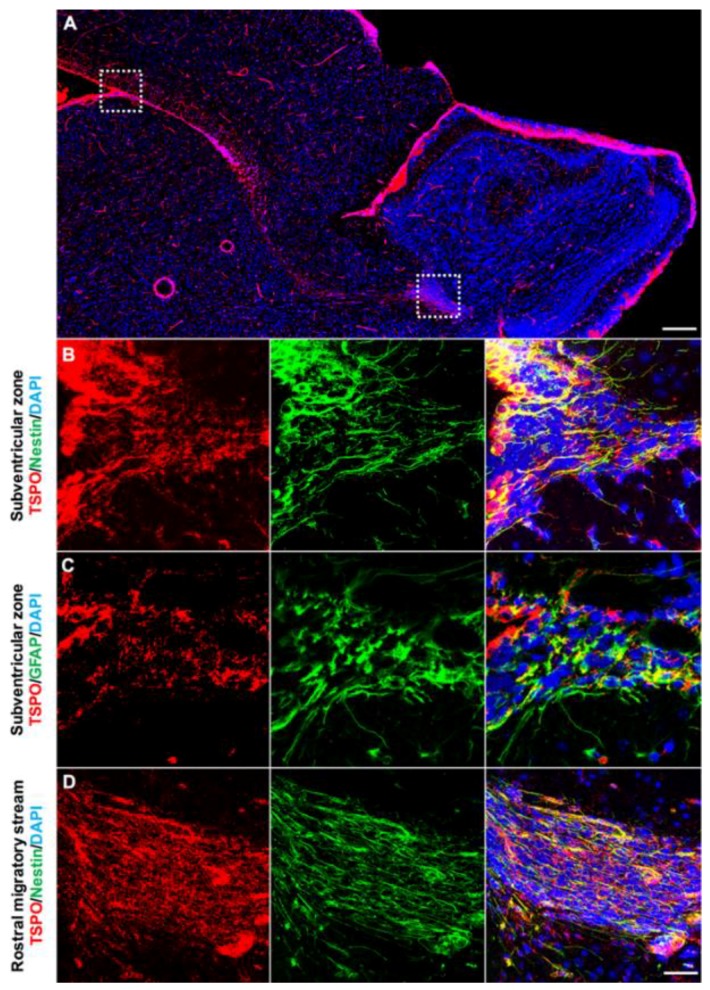
TSPO is expressed in the neurogenic regions of the subventricular zone and the rostral migratory stream (RMS). (**A**) Strong TSPO immunofluorescence (red) is observed in the subventricular zone adjacent to the ependymal lining of the ventricles, and extends through the RMS to the neurogenic niche of the olfactory bulb. (**B**) TSPO expression (red) is observed in Nestin+ neural stem/progenitor cells (green) in the subventricular zone (sagittal view). (**C**) In the RMS, TSPO expression in Nestin+ progenitor cells (green) is also high (sagittal view). (**D**) TSPO expression (red) also colocalizes with GFAP in neural stem/progenitor cells (green) in the subventricular zone (coronal view), confirming the presence of TSPO in neural stem/progenitor cells. Scale bars = 300 µm for (**A**), 20 µm for (**B**–**D**).

We also used double immunofluorescence staining to investigate the presence of TSPO in other neurogenic regions. In sagittal brain sections, prominent TSPO expression was observed in the subventricular zone extending through the RMS (Figure 4A). TSPO immunoreactivity was found in Nestin+ neural stem/progenitors located in the subventricular zone along the ventricular walls adjacent to the ependyma (Figure 4B). This extended through the RMS to the olfactory bulb (Figure 4C). Coronal sections also revealed TSPO expression colocalized with GFAP+ neural stem/progenitors in the subventricular zone (Figure 4C). While neurogenic regions express a variety of markers due to the different subpopulations of newly formed cells, results strongly suggest that TSPO is expressed in neural stem/progenitor cells.

**Figure 5:**
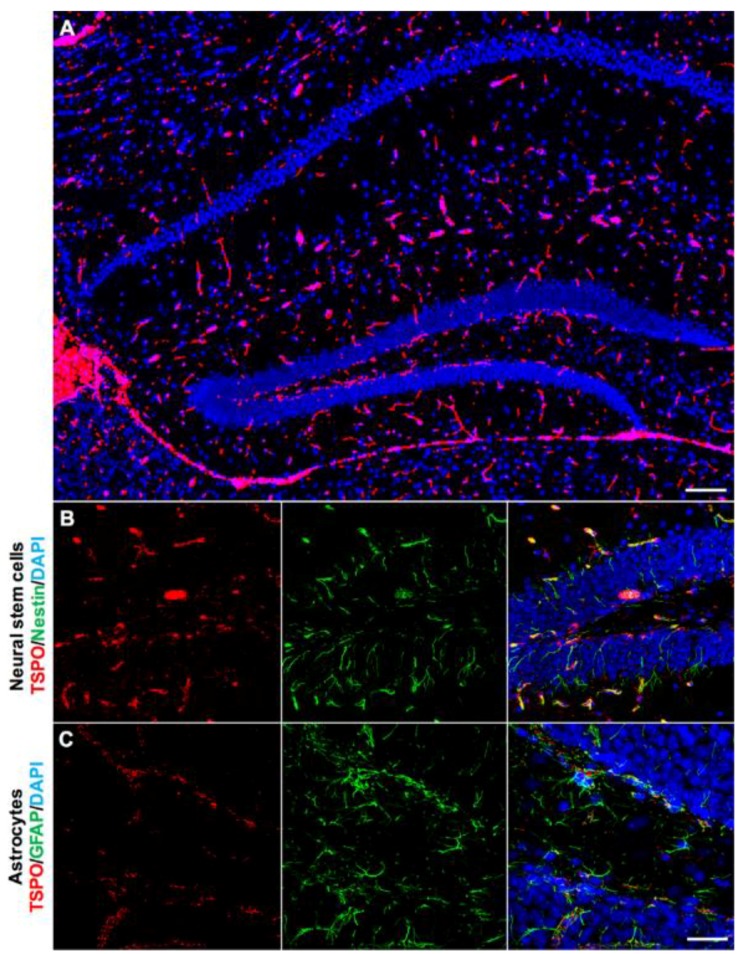
TSPO expression in the hippocampal region of the normal mouse brain. (**A**) TSPO expression (red) and DAPI (blue) to label the distinctive structural features of the region. Strong TSPO immunoreactivity is observed within the subgranular zone of the dentate gyrus and the ependyma. (**B**) TSPO (red) colocalizes at low levels with Nestin+ cell bodies (green), but not processes, in the subgranular zone of the dentate gyrus. (**C**) TSPO expression (red) does not colocalize with GFAP+ astrocytes (green) of the hippocampus, though is present in GFAP+ neural stem/progenitor cells of the subgranular zone, further confirming the expression of TSPO in neural stem/progenitor cells. Scale bar = 300 µm for (**A**), 20 µm for (**B**,**C**).

The hippocampal region of the brain has its own neurogenic niche in the subgranular zone of the dentate gyrus, involved in both adult neurogenesis and gliogenesis. TSPO immunoreactivity was observed throughout the entire hippocampal region, with concentrated expression in the subgranular zone (Figure 5A). Because of the different subpopulations of newly forming cells within neurogenic niches, double immunofluorescence staining was used to confirm that TSPO immunoreactivity colocalizes with Nestin+ (Figure 5B) and GFAP+ neural stem/progenitors of the subgranular zone, though higher expression was observed in GFAP+ cells (Figure 5C). TSPO was not observed on Nestin+ cell processes, rather only at low levels in the cell soma (Figure 5B). 

**Figure 6:**
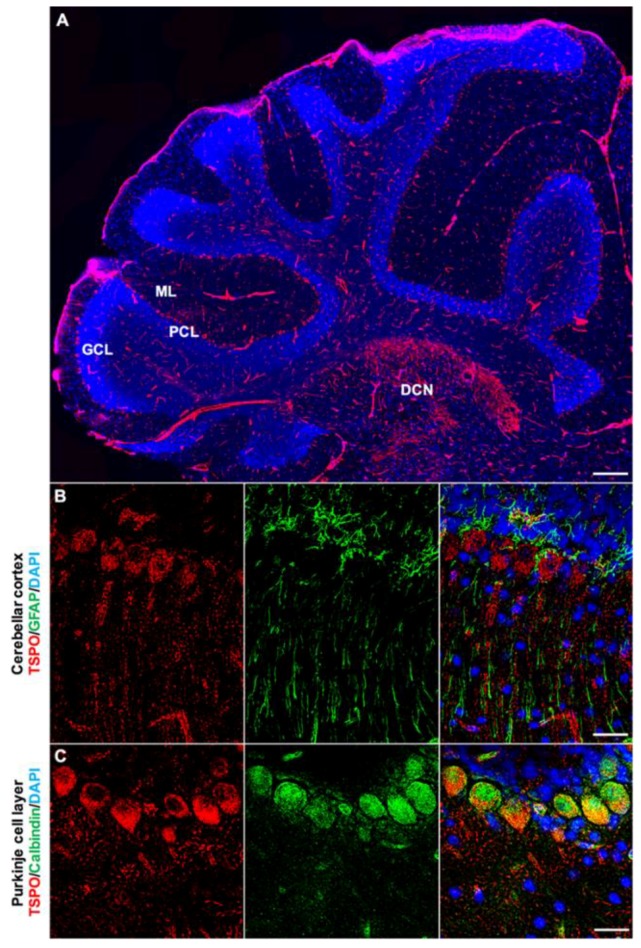
TSPO expression in the cerebellum of the normal mouse brain. (**A**) Sagittal view of TSPO expression (red) across the cerebellar region. Structural features of the region highlighted with DAPI (blue) to delineate cerebellar cortical layers. TSPO is expressed in the Purkinje cell layer and the molecular layer. Also of note is salient TSPO expression in the deep cerebellar nuclei. (**B**) Immunofluorescence staining with TSPO (red) and GFAP (green) in the Purkinje cell layer. TSPO is clearly discernible in Purkinje cells and the molecular layer, which contains dendritic projections. GFAP+ astrocytes/radial glia are also found in this region, though do not express TSPO. (**C**) Calbindin (green) and TSPO (red) immunostaining confirms significant colocalization of TSPO in the perinuclear region of Purkinje cells. Scale bars = 300 µm for (**A**), 20 µm for (**B**,**C**). ML = molecular layer, PCL = Purkinje cell layer, GCL = granule cell layer, DCN = deep cerebellar nuclei.

TSPO is also expressed in the cerebellar cortex, with clearly discernible expression in the Purkinje cell layer and molecular layers of the cerebellar cortex (Figure 6A,B). Concentrated TSPO expression was also observed in the deep cerebellar nuclei, which was found across all sagittal sections obtained (Figure 6A). The presence of TSPO in Purkinje cells was confirmed with double immunofluorescence staining using an anti-Calbindin antibody (Figure 6C), and was predominantly found in the perinuclear region of the Purkinje cell soma. Salient TSPO expression was also observed extending into the molecular layers, indicating the presence of TSPO in the dendritic branches of Purkinje cells. The punctate staining pattern of TSPO was observed in the Purkinje cell soma, as well as throughout the molecular layer, consistent with mitochondrial localization of TSPO. Endothelial cells across the region strongly expressed TSPO, whereas microglia demonstrated no colocalization. Astrocytes were strongly present in the Purkinje cell layer, though GFAP+ astrocytes/radial glia were not positive for TSPO (Figure 6B).

Combined, the presence of TSPO in Purkinje cells, neural stem/progenitor cells, and vascular endothelial cells highlights the diverse range of cell populations where TSPO is distributed in the normal mouse brain. A more comprehensive, semi-quantitative assessment of TSPO localization and relative expression in the aforementioned cell types across brain regions can be found in Table 1.

## 3. Discussion

Constitutive expression of TSPO is high in most peripheral organs, including the adrenals, kidney, spleen, and liver, but low and often below detection limits in the normal healthy brain [3,47,48,49]. However, following injury or during actively progressing tissue pathology, high levels of TSPO can be found in the brain. The inducible TSPO expression that closely follows the distribution pattern of activated microglia rather than astrocytes [8,10,11,50,51] has been the rationale for the use of TSPO ligands for the molecular imaging of active brain pathology irrespective of the primary disease cause [2].

The question of constitutive TSPO has recently become more important in the context of new TSPO ligands that show relatively high signals in the normal brain despite the near absence of expressed TSPO in the brain [47,48], as well as recent observations in experimental and clinical studies reporting reduced levels of TSPO [18,20]. While there is broad consensus that the cellular source of induced TSPO expression in the diseased brain is located in activated microglia [8,10,11,50,51], the distribution of constitutively expressed TSPO has not been fully validated. To this end, we have previously generated a viable global TSPO knockout mouse model to validate the selectivity and specificity of TSPO ligands and antibodies [3]. The current study extends on earlier work by provision of a comprehensive map of TSPO expression in different regions and cell types of the normal mouse brain. Notably, we demonstrate TSPO expression in vascular endothelial cells that gives rise to a punctate immunohistochemical TSPO staining pattern throughout the brain parenchyma, consistent with a mitochondrial localization. No clearly discernible TSPO expression was observed in astrocytes, microglia or oligodendrocytes across the brain. We have demonstrated high levels of TSPO in the glomerular and olfactory nerve layers of the olfactory bulb, and the choroid plexus. Relatively higher TSPO expression compared to the whole brain was also found in regions of neurogenesis; the subventricular zone and the RMS, the subgranular zone of the dentate gyrus, as well as the Purkinje cell layer of the cerebellum.

The observed immunostaining pattern of TSPO expression across brain regions is consistent with previous autoradiographic studies of the rodent brain, including salient expression in the olfactory bulb, ependymal cells of ventricles, and the choroid plexus but generally no TSPO ligand binding above non-specific background in normal white and grey matter [22,23]. More recently, Mirzaei et al. (2016) used autoradiography to depict TSPO binding sites in the normal mouse brain which is high in the olfactory bulb and cerebellar cortex [12], consistent with our immunohistochemical staining. Examination of the cellular distribution of TSPO confirmed that the major cell type expressing TSPO are vascular endothelial cells, consistent with previous reports in both the normal and pathological brain [12,18,34,43]. The other major cell type of TSPO expression are ependymal cells. Along with tanycytes, these are specialized glial cells [52], lining the brain and spinal cord ventricular system to maintain and filter cerebrospinal fluid, as well as support subventricular neural stem cell niches [53,54]. Peripheral immune cells can migrate from the periphery to the brain parenchyma through the endothelial blood-brain-barrier, but also through the choroid plexus, part of the circumventricular system [55]. The low-level constitutive expression of TSPO is most discernible in regions where immune cell trafficking into the parenchyma occurs, involving cells with mitotic turnover and proliferative capacity [56], consistent with the putative function of TSPO in cellular energy metabolism [5,57].

By extension, we also observed strong TSPO expression in neurogenic regions, i.e., zones with proliferating precursor cells. TSPO expression was observed in the olfactory bulb subependymal zone, the subventricular zone, and throughout the RMS, all of which uniquely express a diverse range of neural stem cell markers. TSPO in the olfactory bulb subependymal zone does not colocalize strongly with GFAP positive cells, most likely due to the predominance of more mature developing neurons. In the subventricular zone, TSPO colocalizes with GFAP positive and Nestin positive cells, which extends throughout the RMS. While GFAP is used primarily as a mature astrocytic marker, GFAP is also a known marker of neural stem/progenitor cells of the SVZ [58]. Nestin positive cells of the hippocampal subgranular zone did not strongly colocalize with TSPO. The complex nature of neural stem cells means that no one marker is sufficient to stain all progenitor types, which may explain the inconsistent expression of TSPO in neural stem/progenitor cells. However, the distinctive expression of TSPO within these neurogenic regions strongly suggests the presence of TSPO in certain subpopulations of neurogenic cell types. More detailed future studies on TSPO in the adult neurogenic niche should be conducted.

We also explored the expression of TSPO in other major cell types of the brain, i.e., astrocytes, pericytes and microglia. The pericyte/smooth muscle marker PDGFRβ was only found to colocalize with TSPO in large blood vessels, suggesting a dependence on the size of the blood vessel, which requires further studies. No TSPO expression was observed in astrocytes and their endfeet surrounding the perivascular region and associating with endothelial cells. The expression of TSPO in astrocytes has been debated, with some groups demonstrating TSPO colocalization in astrocytes [43], with increases in reactive astrocytes [45] or no such colocalization [14,41].

Our observations confirm no clearly discernible TSPO expression in microglia of the normal mouse brain. While glial TSPO is due to de novo expression and, thus, only gives rise to increases in TSPO binding signals in molecular imaging studies, the constitutive parenchymal-vascular TSPO expression may also be expected to decrease, either due to a down-regulation or a rarefication of the vascular bed. Notably, in conditions of non-destructive and non-progressive subtle pathology with apparent reduction in TSPO expression, and without a significant presence of activated microglia, we suggest that a signal reduction should not be interpreted as a reduced expression by microglia, since the resting microglia in these conditions do not show any TSPO expression that could be reduced further. The widely distributed, albeit sparse, parenchymal-vascular expression of TSPO has previously prompted the development of different approaches to PET quantification [30,31,32,33,34,35], though it remains to be established when and how TSPO imaging quantification methods need to account for variations in the small vasculature-associated baseline expression of TSPO.

In contrast to the TSPO expression in mitotically active and proliferative cell types, the constitutive TSPO expression in cerebellar Purkinje cells, a population of large GABA-ergic mature neurons is unexpected, though previously observed by Wang et al. (2012) [46]. Validated by the absent staining in the TSPO knockout animals, TSPO expression is clearly present in the perinuclear region and extending into dendrites of cerebellar Purkinje cells. TSPO expression in mature neurons has also previously been reported by other groups [18], despite our current finding that NeuN positive mature neuronal populations are immunonegative for TSPO. The finding of TSPO expression in both mitotically active cells as well as post-mitotic, end-differentiated cell populations including mature neurons, hints at additional unknown roles for TSPO in the cellular functioning of highly complex cells and the potentially underlying differentiation of their mitochondrial apparatus [59].

Our findings support the existing notion that resting glial cells do not express TSPO, but that there is sparse, low-level expression of TSPO in the normal brain parenchyma that locates with mitochondria of endothelial cells. Such constitutive expression, though compared to the constitutive expression levels in other organs is very low, is expected to give rise to a low baseline signal in TSPO molecular imaging studies. Reductions in signal compared to baseline have recently been reported in experimental and clinical studies. We speculate that reduced levels of TSPO may reflect changes in the state of endothelial vasculature due to developmental and/or disease related pathology. Though the reported constitutive vascular-endothelial TSPO expression is small in comparison to the levels of TSPO observed in diseased tissue with activated microglia, it may well affect quantitative measurements of TSPO, notably if reference tissue regions are chosen from within the same organ. Likewise, the high constitutive TSPO expression in the normal olfactory nerve layer and glomeruli of the olfactory bulb, ependymal layers and choroid plexus needs to be kept in mind when identifying disease-affected areas in the vicinity of these structures.

In summary, the viable TSPO knockout mouse model provides a robust validating negative control and reference tissue against which to interpret PET imaging studies using TSPO ligands and establish selectivity. The unexpected presence of TSPO selectively in cerebellar Purkinje cells, but not other mature neurons, suggests functions of the mitochondrial TSPO beyond its known role in energy metabolism or hormonal synthesis.

## 4. Materials and Methods

### 4.1. Animals and Tissue Preparation

Six week old wild-type (WT) C57BL/6 mice were obtained from the Animal Resource Centre (Western Australia). TSPO knockout mice with C57BL/6 genetic background (C57BL/6-*Tspo^tm1GuWu(GuwiyangWurra)^*) were bred at the Australian Nuclear Science and Technology Organisation (ANSTO) as previously described [3]. All animal procedures were approved by the University of Sydney Animal Ethics Committee and the ANSTO Animal Care and Ethics Committee (P301, 31 March 2017). All procedures were in accordance with the Australian Code of Practice for the Care and Use of Animals for Scientific Purposes (8th edition, 2013). Three WT and TSPO knockout mice were sacrificed using isofluorane overdose and exsanguination. Brains were removed and snap frozen in liquid nitrogen and kept at −80 °C until sectioning. Coronal and sagittal brain sections were cut at 10 µm thickness on a cryostat (Leica CM3050) at −20 °C according to the Paxinos and Franklin Mouse brain atlas [60]. Coronal tissue sections were collected from the olfactory bulb, the subventricular zone, the expanse of the hippocampal region and the cerebellum and brainstem. Sagittal sections were collected from lateral 1.32mm to lateral 0.36mm. Sections were mounted onto Poly-l-lysine slides (Thermo Scientific, Rockford, IL, USA) and slides were stored at 4°C until subsequent use.

### 4.2. Immunohistochemistry/Immunofluorescence 

Immunohistochemistry was performed as previously described in Banati et al. (2014) [3]. Tissue sections were fixed with 3.7% formaldehyde in PBS for 5 min, then permeabilized with ice-cold acetone. Non-specific antibody binding was blocked with 5% goat serum and 5% bovine serum albumin (BSA) in PBS. Sections were incubated with a TSPO rabbit monoclonal antibody (ab109497, Abcam) at 4 °C overnight, then incubated with a secondary biotinylated goat anti-rabbit antibody at room temperature for 1 h using the Vectastain ABC kit (PK-4001, Vector Laboratories, Burlingame, CA, USA). Sections were then incubated with the ABC solution, and developed using a metal-enhanced 3,3′-diaminobenzidine tetrahydrochloride DAB substrate kit (#34065, Thermo Scientific). Sections were then dehydrated in a graded series of ethanol followed by xylene, and were then mounted and coverslipped in DPX mounting medium (Sigma-Aldrich, St. Louis, MO, USA).

For triple immunofluorescence staining, all sections were labelled with a TSPO rabbit monoclonal antibody (ab109497, Abcam, Cambridge, UK) and one of the following antibodies: anti-CD11b (rat; MCA711, Bio-Rad, Hercules, CA, USA), anti-CD31 (rat; MCA2388T, Bio-Rad), anti-GFAP (rat; 13-0300, Thermo Fisher Scientific, Rockford, IL, USA), anti-PDGFRβ (rat; ab91066, Abcam), anti-MBP (rat; ab7349, Abcam), anti-Nestin (rat; ab81462, Abcam), anti-NeuN (mouse; MAB377, Merck Millipore, Billerica, MA, USA) and anti-Calbindin (mouse; ab82812, Abcam) at 4 °C overnight (refer to Supplementary Table S1 for all antibody details). Sections were then incubated with Alexa Fluor 594-conjugated anti-rabbit IgG (ab150080, Abcam), Alexa Fluor 488-conjugated goat anti-rat IgG (ab150165, Abcam), Alexa Fluor 488-conjugated goat anti-mouse IgG (ab150117, Abcam) or Alexa Fluor 488-conjugated goat anti-chicken IgY (ab150169, Abcam) at room temperature for 1 h. Sections were mounted with ProLong Diamond Antifade Mountant with DAPI (Thermo Fisher Scientific) and coverslipped. Immunostaining on negative control sections of normal mice without the primary antibodies, and sections of TSPO knockout tissue, was performed for each antibody listed.

### 4.3. Microscopy 

Scans of whole tissue sections were obtained using the Invitrogen EVOS FL Auto Imaging System (Thermo Fisher Scientific). To determine cellular colocalization and abundance of TSPO, higher magnification microscopy was performed under a BX61WI Olympus microscope, and images were acquired with a digital camera (CoolSNAP, Photometrics, Tucson, AZ, USA) and the Image InVivo program (Photometrics). Z-stacks were acquired with 40X and 100X water immersion objectives and deconvolved using AutoQuant X3 software (Media Cybernetics, Rockville, MD, USA), then further processed with ImageJ software (NIH, Baltimore, MD, USA). Images were acquired under the same conditions with exposure times and gain kept constant.

### 4.4. Semi-Quantification of TSPO Intensity and Colocalization

TSPO localization and relative expression levels across brain regions and major cell types were assessed semi-quantitatively. TSPO colocalization with cell types was determined using ImageJ analysis of high magnification double immunofluorescence images. TSPO (red channel) combined with all other cell markers (green channels) were merged using ImageJ, and degree of overlap (yellow pixels) was determined for each cell type. A grading system was used, ranging from an absence of staining (−) to significant staining across regions and cell types (++). Colocalization of TSPO with CD11b was further examined with z-stack movies.

## Figures and Tables

**Table 1:** Semi-quantitative assessment of TSPO immunoreactivity in various cell types of the brain by region.

Brain Region	Endothelial Cells (CD31+)	Microglia (CD11b+)	Astrocytes/NSC (GFAP+)	NSC (Nestin+)	Pericytes (PDGFRβ+)	Purkinje Cells (Calbindin+)	Oligodendrocytes (MBP+)	Mature Neurons (NeuN+)	Ependyma
Olfactory bulb	++	−	++	++	+	−	−	−	−
SVZ	++	−	++	++	+	−	−	−	−
Cortex	++	−	−	−	+	−	−	−	−
Choroid plexus/ventricular system	−	−	−	−	−	−	−	−	++
Hippocampus	++	−	+	+	+	−	−	−	−
Striatum/thalamus	++	−	−	−	+	−	−	−	−
Cerebellum/brainstem	++	−	−	−	+	++	−	−	−

(−) indicates no colocalization, (+) weakly labelled, or (++) high colocalization. NSC = neural stem cells, SVZ = subventricular zone.

## Supplementary Materials

Supplementary materials can be found at http://www.mdpi.com/1422-0067/19/9/2707/s1.

## Abbreviations

TSPO	Translocator protein
WT	Wild-type
BSA	Bovine serum albumin
DAB	3,3′-diaminobenzidine
DAPI	4’,6-diamidino-2-phenylindole
RMS	Rostral migratory stream
SVZ	Subventricular zone
NSC	Neural stem cells
PET	Positron emission tomography
ML	Molecular layer
PCL	Purkinje cell layer
GCL	Granule cell layer
DCN	Deep cerebellar nuclei
