# Supplementary material for: Cellular Sources and Regional Variations in the Expression of the Neuroinflammatory Marker Translocator Protein (TSPO) in the Normal Brain
Source: Int J Mol Sci. 2018 Sep 11;19(9):2707. doi: 10.3390/ijms19092707 (PMC6163555; doi:10.3390/ijms19092707)
Supplement: Supplementary file 1 [file ijms-19-02707-s001.zip › ijms-346241-SM-for proofreading.pdf]

Supplementary Information

Cellular sources and regional variations in the expression of the neuroinflammatory marker Translocator Protein 18 kDa (TSPO) in the normal brain

Calina Betlazar [1,2], Meredith Harrison Brown [1,2], Ryan J. Middleton [1], Richard Banati [1,2] and Guo-Jun Liu [1,2,*]

1 Australian Nuclear Science and Technology Organisation, New Illawarra Road, Lucas Heights, NSW 2234, Australia
2 Discipline of Medical Imaging & Radiation Sciences, Faculty of Medicine and Health, Brain and Mind Centre, University of Sydney, 94 Mallett Street, Camperdown, NSW 2050, Australia
* Correspondence: calinab@ansto.gov.au; gdl@ansto.gov.au; rib@ansto.gov.au

Supplementary Materials and Methods

Supplementary Table S1. Primary antibodies used for immunohistochemistry/fluorescence staining in this study. Specificity of the TSPO antibody has been validated in a global TSPO knockout mouse model [1]. Note that GFAP was used to detect mature astrocytes as well as neural stem/progenitors in neurogenic regions.

Target	Description	Dilution	Supplier	ID #
TSPO	Rabbit monoclonal	1:400	Abcam	ab109497
CD11b	Rat monoclonal	1:200	Bio-Rad	MCA711
CD31	Rat monoclonal	1:400	Bio-Rad	MCA2388T
Nestin	Rat monoclonal	1:200	Abcam	ab81462
PDGFRß	Rat monoclonal	1:50	Abcam	ab91066
GFAP	Rat monoclonal	1:200	Thermo Fisher	13-0300
Calbindin	Mouse monoclonal	1:100	Abcam	ab82812
NeuN	Mouse monoclonal	1:100	Merck	MAB377
MBP	Rat monoclonal	1:100	Abcam	ab7349

Supplementary Results

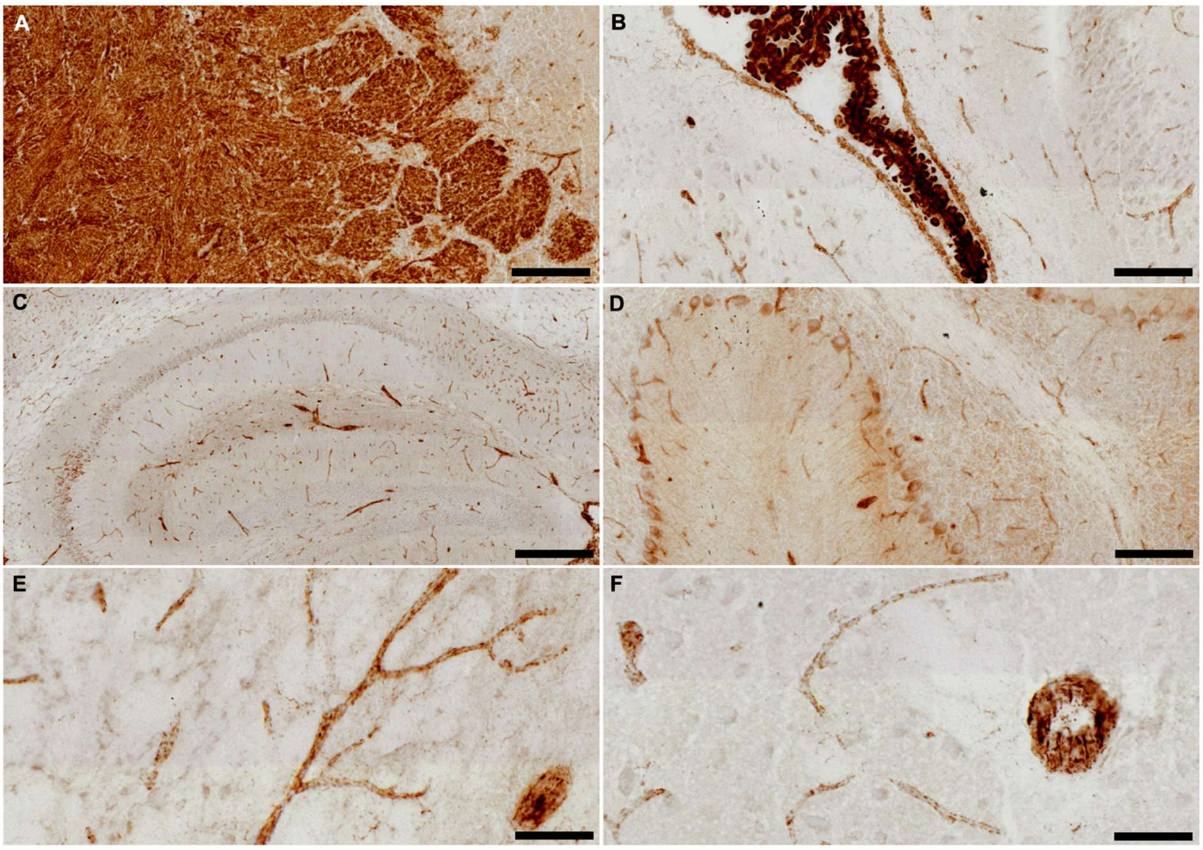

Supplementary Figure S1. TSPO distribution visualised with DAB immunohistochemistry using a validated TSPO antibody (#ab109497, Abcam). **(A)** Nerve trunks in the olfactory bulb; **(B)** Choroid plexus; **(C)** Hippocampus; **(D)** Purkinje neurons in cerebellum; **(E)** small blood vessels and capillaries; **(F)** artery and capillaries. Scale bars = 100µm for A, B, C and D; scale bars = 40µm for E and F.

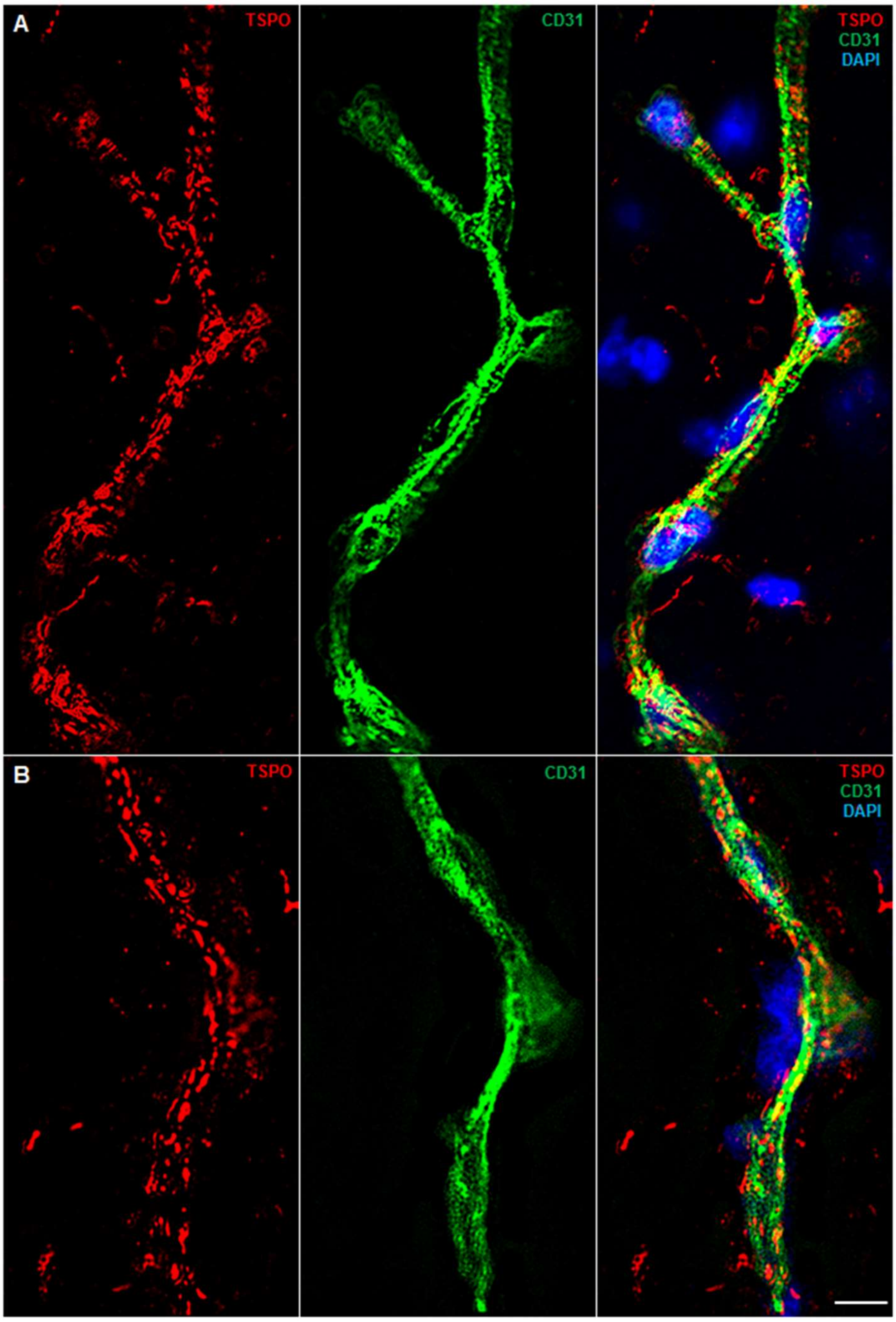

Supplementary Figure S2. TSPO strongly localises in vascular endothelial cells of the brain. **(A, B)** CD31+ blood vessels across all brain regions ubiquitously express TSPO. Note the punctate staining pattern of TSPO, indicative of mitochondrial localisation. Scale bar = 20μm.

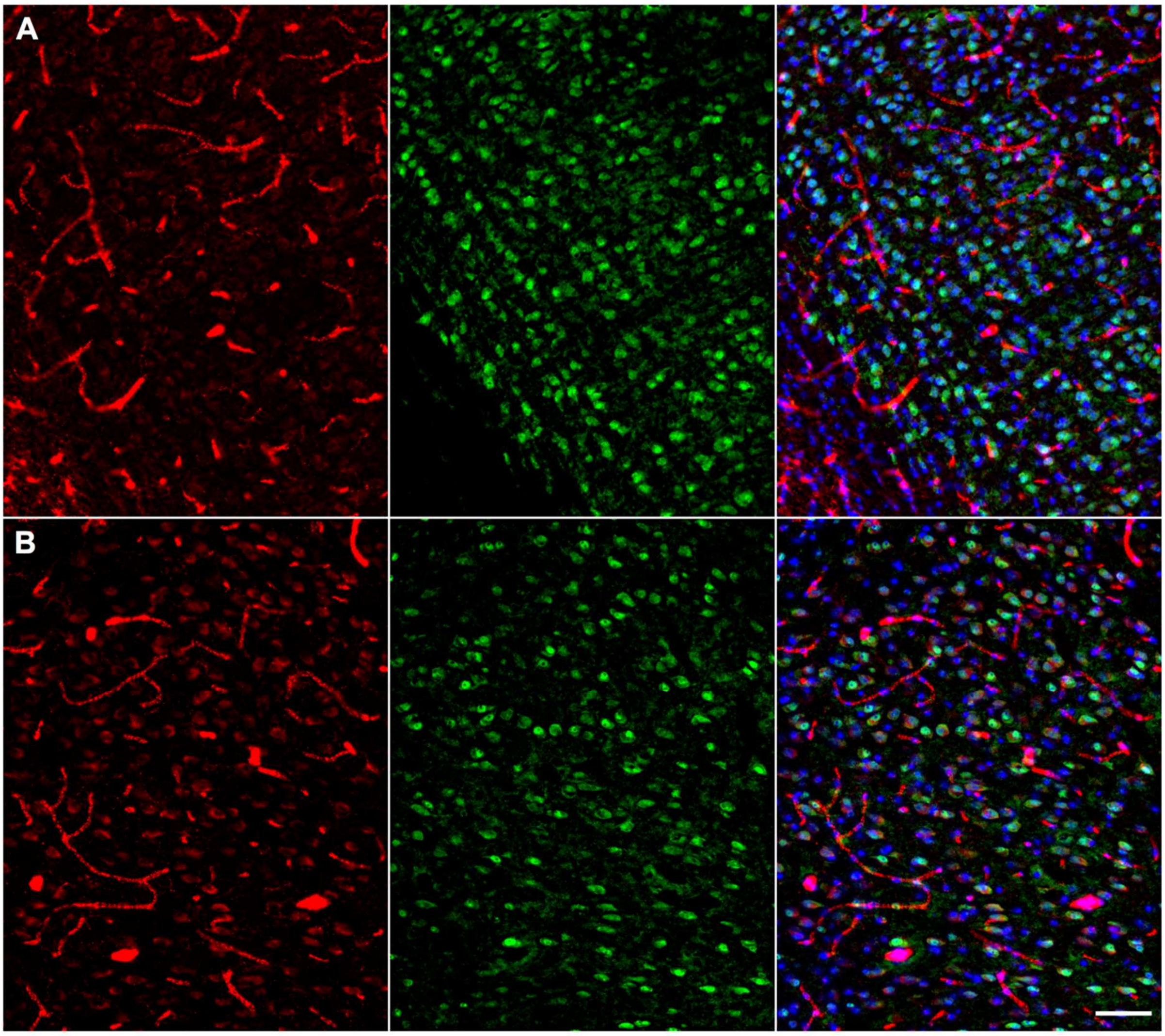

Supplementary Figure S3. Mature neuronal populations do not express TSPO. NeuN+ neurons of the cerebral cortex **(A)** and thalamus **(B)** are not TSPO positive. Scale bar = 20μm.

Supplementary References

1. Banati RB, Middleton RJ, Chan R, Hatty CR, Kam WW, Quin C, et al. Positron emission tomography and functional characterization of a complete PBR/TSPO knockout. Nat Commun. 2014;5:5452.
